# A difference in larval mosquito size allows a biocontrol agent to target the invasive species

**DOI:** 10.1002/ece3.10294

**Published:** 2023-07-10

**Authors:** Marie C Russell

**Affiliations:** ^1^ Department of Life Sciences Imperial College London Ascot UK

**Keywords:** biocontrol, cyclopoid copepods, invasion ecology, mosquito‐borne disease, predator–prey interactions

## Abstract

As the global temperature rises in the coming decades, *Aedes albopictus* is expected to invade and establish in South East England, where *Culex pipiens* is currently the most common native mosquito species. Biocontrol measures that use local cyclopoid copepods against *Ae. albopictus* may be compromised if the copepods prefer alternate *Cx. pipiens* prey. In this study, I assessed the predation efficiency of *Megacyclops viridis* copepods against *Ae. albopictus* larvae from France and larvae that hatched from egg rafts of *Cx. pipiens* collected in South East England. The experiments were conducted at 15 and 25°C, which are representative of present and future summer temperatures in South East England. *Ae. albopictus* larvae that survived the course of the experiment in the predator‐absent controls were significantly smaller than *Cx. pipiens* larvae that survived in the absence of predation. The background mortality of *Cx. pipiens* larvae increased with the 10‐degree increase in temperature, and the smaller size of surviving *Cx. pipiens* larvae at 25°C, relative to survivors at 15°C, suggests that larger *Cx. pipiens* larvae were more likely to die at the higher temperature setting. Across all experimental treatments, the ratio of copepod body length to mean prey length, based on larval lengths of survivors from the corresponding predator‐absent controls, was a significant predictor of the copepod's predation efficiency. Adding temperature setting to the predation efficiency model as a predictor did not improve model fit. Within the mixed prey treatments, the predation efficiency of *M. viridis* was 34.5 percentage points higher against *Ae. albopictus* prey than against *Cx. pipiens* prey. The higher predation efficiency that *M. viridis* exhibited against invasive *Ae. albopictus* prey, likely due to the smaller size of these larvae, supports the future use of *M. viridis* as a biocontrol agent in the United Kingdom.

## Graphical abstract

The ratio of copepod body length to mean mosquito prey length was a significant predictor of the copepod's predation efficiency. Within the mixed prey treatments, the predation efficiency of *Megacyclops viridis* was 34.5 percentage points higher against *Aedes albopictus* prey than against *Culex pipiens* prey. The higher predation efficiency that *M. viridis* exhibited against invasive *Ae. albopictus* prey, likely due to the smaller size of these larvae, supports the future use of *M. viridis* as a biocontrol agent in the United Kingdom.

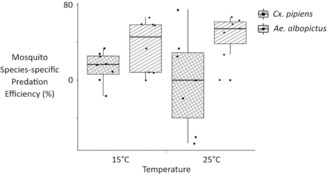

## 1 INTRODUCTION

Invasive *Aedes albopictus* mosquitoes lay desiccation‐resistant eggs in artificial containers, such as tyres (Benedict et al., 2007; Eritja et al., 2005; Juliano & Lounibos, 2005; Lounibos, 2002; Medlock et al., 2006), and are likely to establish in South East England within the next three to six decades (Kraemer et al., 2019; Metelmann et al., 2019; Proestos et al., 2015). According to the European Centre for Disease Prevention and Control, *Ae. albopictus* populations have already established throughout Italy and most of southern France (ECDC and EFSA, 2023). Although this species has not yet established in the United Kingdom, it has been introduced in Kent, a coastal county in South East England (Vaux et al., 2019). In North America, *Ae. albopictus* is expanding its range into Canada, where it is considered established in Windsor, Ontario (Giordano et al., 2020). Predation by cyclopoid copepods has previously been identified as a method for controlling *Ae. albopictus* populations in Europe (Baldacchino et al., 2015), but more research is needed to assess the efficacy of copepod biocontrol agents against *Ae. albopictus* in the presence of native mosquito prey species.

*Culex pipiens* is one of the most common species of mosquito in England, where it has been found in the shallow water of marshes, as well as in artificial containers, including tyres (Chapman et al., 2017; Golding et al., 2015). A field study conducted in Italy found that 67% of all *Ae. albopictus* larvae shared their larval habitats with *Cx. pipiens* (Carrieri et al., 2003). *Aedes* larvae have previously displayed higher motility than *Culex* larvae (Dieng et al., 2003; Kesavaraju et al., 2011), and higher larval activity has been associated with greater vulnerability to predation (Grill & Juliano, 1996). Cyclopoid copepods are likely to respond to higher motility because they feed on live prey by using mechanoreception to detect hydrodynamic disturbances (Awasthi et al., 2012; Roche, 1990). This is consistent with the findings of multiple studies in which cyclopoid copepods have demonstrated a preference for *Aedes* prey, rather than *Culex* (Dieng et al., 2003; Pauly et al., 2022; Soumare & Cilek, 2011). However, prey size is also an important stage‐dependent trait that varies across species and can influence vulnerability to predation (Alto et al., 2009). For example, cyclopoid copepods have been shown to prefer smaller species of rotifer prey (Lapesa et al., 2002), and copepod‐imposed mortality among fish larvae is inversely related to larval age and body length (Kumar et al., 2012).

By 2050, it is predicted that London's climate will resemble that of present‐day Barcelona (Bastin et al., 2019). According to the temperature‐size rule of ‘hotter is smaller’, ectotherms are likely to exhibit phenotypic plasticity, in which higher temperatures during development produce smaller adults (Kingsolver & Huey, 2008). In addition, among ectothermic aquatic organisms, climate change is likely to result in an increase in the proportion of small‐sized species and young age classes and a decrease in size‐at‐age (Daufresne et al., 2009). While one meta‐analysis found that the attack rate of copepod predators increases with temperature (Kalinoski & DeLong, 2016), another meta‐analysis of functional response curves suggests that the increase in attack rate due to temperature is less steep than one would expect based on metabolic theory (Rall et al., 2012). For sit‐and‐wait predators, such as cyclopoid copepods, the effect of temperature on predation is driven mainly by prey velocity (Dell et al., 2014). The lack of a significant effect of temperature on the attack rate of *Megacyclops viridis* copepods against *Ae. albopictus* prey, based on previous research (Russell et al., 2021), suggests that the velocity of newly hatched *Ae. albopictus* larvae does not change dramatically over a temperature range of 15–25°C.

In this study, I assess the predation efficiency of *M. viridis* copepods from Surrey, UK, which have previously been recommended as suitable biocontrol agents against invasive mosquitoes (Russell et al., 2021). The prey organisms include progeny of wild‐caught *Cx. pipiens* from Berkshire, UK and *Ae. albopictus* from Montpellier, France. A previous field survey of artificial tyre microhabitats in London and Berkshire, UK found that temperatures taken from May through September of 2018, between the hours of 11 am and 5 pm, ranged from 9 to 25°C (Russell et al., 2021). For this study, the temperatures of 15 and 25°C were chosen from the upper end of the 9–25°C range to account for the effects of climate change expected to occur in the next three to six decades.

## 2 MATERIALS AND METHODS

### 2.1 Collection of *Cx. pipiens* egg rafts from gravid females

Adult gravid female *Culex* mosquitoes were collected on the 24th of June and the 13th of July 2019 from Ascot, Berkshire, UK near Silwood Lake (N 51°24.876′, W 0°39.045′) using a CDC gravid trap from the John W. Hock company (Model 1712). The trap was set on the preceding evening with 4 L of hay infusion in a 34 × 24.5 × 17 cm black plastic container as the attractant, and a battery‐operated fan was used to capture females during oviposition. Once captured, each gravid *Culex* female was aspirated into a 25 × 25 × 25 cm cage and provided with 10% sucrose solution. A black plastic cup, containing 100 mL spring water, 15 mg spirulina, 33 mg Tetramin® fish food, 33 mg Russell Rabbit Tasty Nugget® rabbit pellets and 33 mg powdered liver, was also provided for oviposition. The gravid *Culex* females were held in individual cages at 20 ± 1°C in a 12:12 light/dark cycle with 70% relative humidity until they laid an egg raft. After oviposition, the adult females were frozen at −20°C and identified by morphology as *Cx. pipiens*/*torrentium*, according to an identification key for British mosquitoes (Cranston et al., 1987). Because it is impossible to distinguish between *Cx. pipiens* and *Cx. torrentium* by morphology, each female was stored at −80°C, and eventually a portion of the 3′ region of the mitochondrial COI gene was sequenced to make a genetic identification (Hesson et al., 2010).

### 2.2 Temperate *Ae. albopictus* colony care

A colony of *Ae. albopictus* mosquitoes (original collection Montpellier, France 2016, obtained through Infravec2) was maintained at 27 ± 1°C, 70% relative humidity, and a 12:12 light/dark cycle. Larvae were fed fish food (Cichlid Gold Hikari®), and adults were given 10% sucrose solution and horse blood (First Link Ltd) administered through a membrane feeding system (Hemotek®).

### 2.3 *M. viridis* copepod cultures

Adult gravid female cyclopoid copepods were collected in May of 2019 from the edge of Longside Lake in Egham, Surrey, UK (N 51°24.298′, W 0°32.599′), and separate cultures were started from each gravid female. Each culture was kept in a 2.6 L container (24.8 × 18 × 9.3 cm) of spring water (Highland Spring) at a 12:12 light/dark cycle and 20 ± 1°C. *Chilomonas paramecium* and *Paramecium caudatum* (Sciento) were provided ad libitum as food. Adult copepods were identified as *Megacyclops viridis* (Jurine, 1820) by Dr. Maria Hołyńska from the Museum and Institute of Zoology in Warsaw, Poland.

### 2.4 Experimental design

**FIGURE 1:**
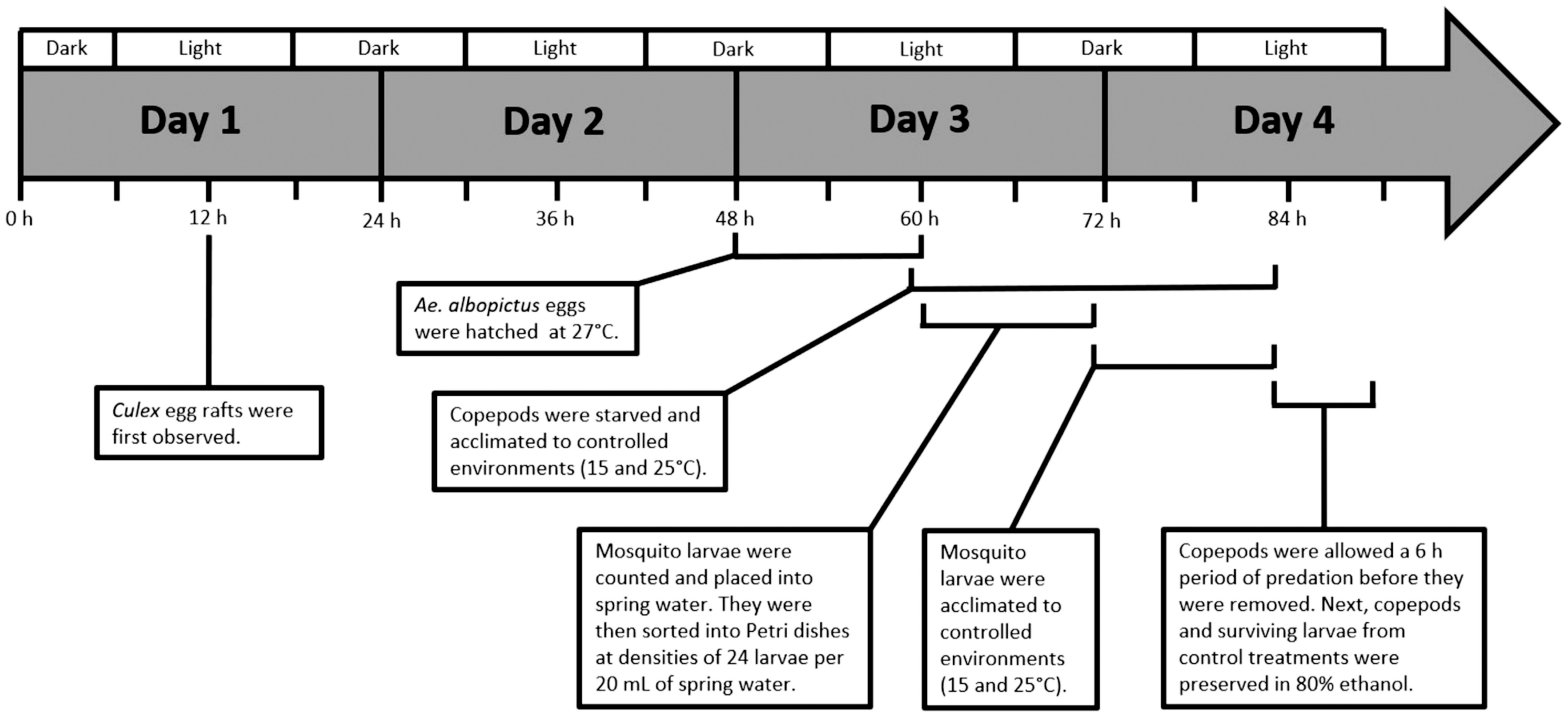
Schedule for predation efficiency experiment with native and invasive mosquito prey.

Cages holding gravid *Culex* females were checked each day at noon for egg rafts. When egg rafts were found, plans were made to hatch the corresponding number of *Ae. albopictus* eggs beginning at midnight, 36 h after finding the *Culex* egg raft (Figure 1). This schedule allowed there to be enough newly hatched larvae of each genus by noon, 48 h after the new *Culex* egg rafts were first observed.

**FIGURE 2:**
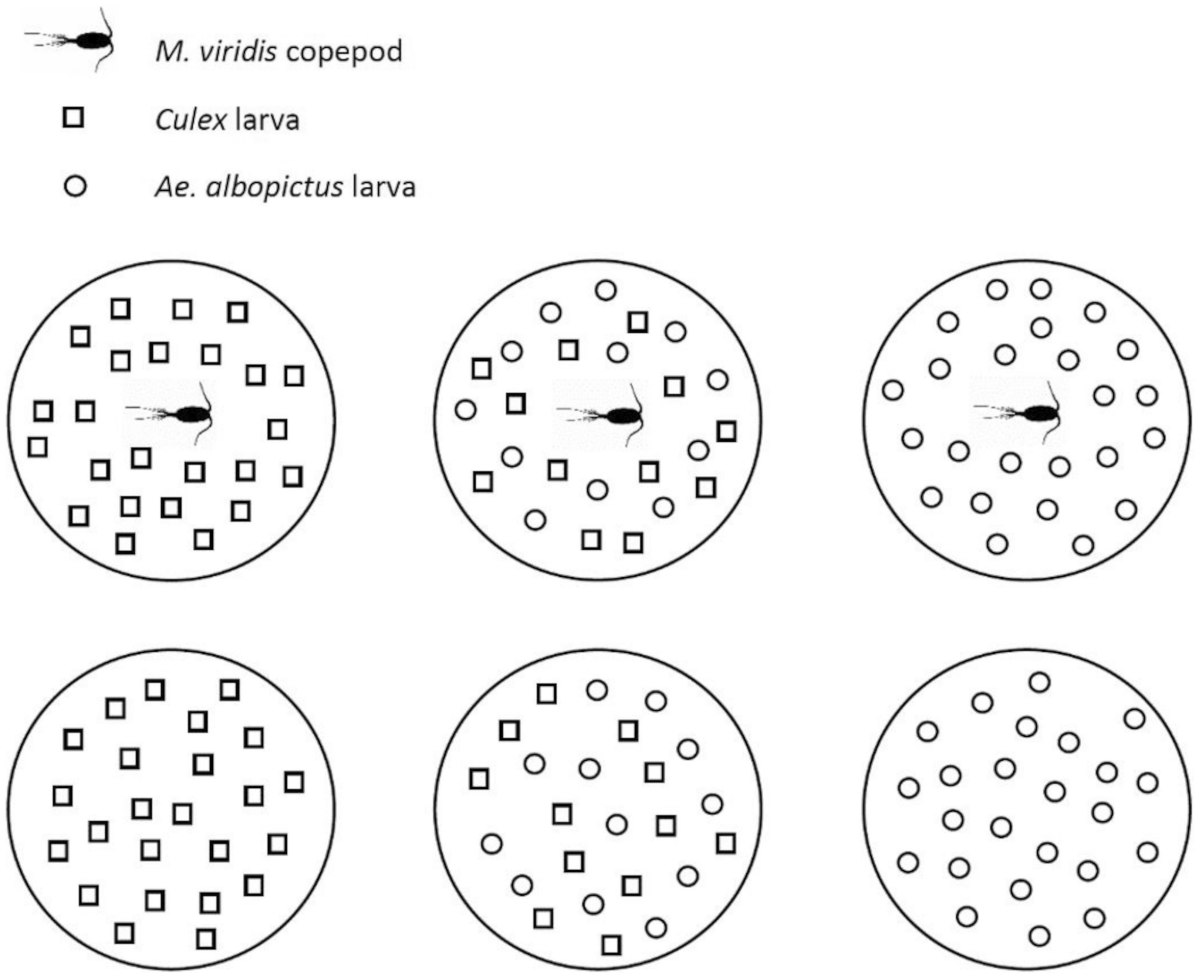
Experimental design repeated 16 times for each combination of *Culex* egg raft (8) and temperature (2). Copepod image source: phylopic.org (CC0 1.0 Universal Public Domain Dedication, creator: Joanna Wolfe).

Adult non‐gravid female *M. viridis* were each placed in a Petri dish holding 20 mL spring water for a 24 h period of starvation and acclimation to two different temperature settings: 15 ± 1 and 25 ± 1°C, both at a 12:12 light/dark cycle. Mosquito larvae were counted and divided into Petri dishes so that one third of the Petri dishes held 24 *Culex* larvae, one third held 12 *Culex* larvae and 12 *Ae. albopictus* larvae, and one third held 24 *Ae. albopictus* larvae (Figure 2); half of these Petri dishes were acclimated to 15 ± 1°C, and the other half were acclimated to 25 ± 1°C. Copepods were introduced to larvae in half of the Petri dishes for a 6 h period of predation, a time period used in previous experiments on this topic (Russell et al., 2021). After the predation period, copepods were removed, and the numbers of surviving larvae were counted and recorded. Copepods and surviving mosquito larvae from the control treatments were stored in 80% ethanol. The procedure was designed so that each treatment replicate with *Culex* larvae was matched with a control that had *Culex* larvae from the same egg raft. Additionally, each treatment replicate with *Ae. albopictus* larvae was matched with a control that had *Ae. albopictus* larvae from the same hatching procedure. The experimental schedule is displayed in Figure 1.

### 2.5 Copepod and mosquito larva length measurements

The body lengths of all copepods, from the front of the cephalosome to the end of the last urosomite, were measured to the nearest tenth of a millimeter using a light microscope (Meiji Techno Co. Ltd) with an ocular scale bar. The body lengths of all surviving mosquito larvae in the control treatments were measured to the nearest hundredth of a millimeter, from the front of the head to the end of the saddle, using a ZEISS AxioCam microscope camera and AxioVision software (4.6.3).

### 2.6 Statistical analyses

All analyses were conducted in R version 4.0.2 (R Core Team, 2020). Pearson's chi‐squared tests were used to determine whether temperature affected the survival of mosquito larvae of each genus in the controls. A linear regression model was fitted to test mosquito genus and temperature as predictors of larval length. Welch two sample *t*‐tests were conducted to determine whether length differed between mosquito genera and to determine whether temperature affected length of surviving larvae within each genus. An additional linear regression model was fitted to test egg raft and temperature as predictors of *Culex* larval length.

Predation efficiency was calculated according to Abbott's formula (Abbott, 1987; Baldacchino et al., 2017):

(1) $$\begin{array}{c} \text{Predation efficiency}\\ =\frac{\text{Number alive in control}-\text{Number alive in treatment}}{\text{Number alive in control}}\;\left(100\right) \end{array}$$

Copepod body mass (mg) was also calculated using an equation from previous studies (Alcaraz & Strickler, 1988; Klekowski & Shushkina, 1966; Novich et al., 2014):

(2) $$\text{Mass}=0.055\times {\text{length}}^{2.73}$$

The mean prey length for each replicate (*n* = 48) was calculated based on the lengths of the surviving larvae in the controls. The predator–prey size ratio was then calculated by dividing the copepod length by the mean prey length. The size ratio values were restricted to two significant figures because the copepod lengths had two significant figures.

A linear regression model was fitted to test copepod length, copepod body mass or copepod‐to‐larva size ratio as predictors of predation efficiency. Temperature, as well as an interaction between temperature and size ratio, were also tested as predictors. Replicates that had >75% larval mortality in the control (*n* = 2) were excluded from predation efficiency analyses. One additional replicate was excluded because the copepod died during the period of predation.

Among replicates with prey of a single mosquito genus (*n* = 29), a Welch two sample *t*‐test was used to determine whether predation efficiency differed significantly (*α* = 0.05) due to the genus of mosquito prey. Within each mixed prey replicate (*n* = 16), predation efficiency was also calculated as two separate values, each specific to a different genus of mosquito prey. One replicate was excluded because zero *Culex* larvae survived in the control. To assess whether the *M. viridis* copepods preferred one type of prey to another, a paired *t*‐test was used to determine whether the differences between mosquito genus‐specific predation efficiencies, within each replicate (*n* = 15), were significantly different than zero.

## 3 RESULTS

According to sequences of 838–854 base pairs in the 3′ region of the mitochondrial COI gene from the eight *Culex* females that provided egg rafts, the *Culex* larvae observed in this experiment belonged to the species *Cx. pipiens* (Hesson et al., 2010). The Basic Local Alignment Search Tool (BLAST) indicated at least 95% query cover and >99% identity, with *E*‐values of 0.0, when each sequence was compared to a *Culex pipiens pipiens* sequence stored in GenBank® (Clark et al., 2016; Table S1).

The probability of survival among *Ae. albopictus* control larvae was 86.8% at 15°C and 86.1% at 25°C and did not differ significantly based on temperature (*p*‐value = .8076). However, among *Cx. pipiens* control larvae, the probability of survival was significantly lower at 25°C, where 48.6% survived, as opposed to 15°C, where 93.8% survived (*p*‐value < .0001). The results of a Welch two sample *t*‐test showed that surviving *Cx. pipiens* control larvae were significantly larger than surviving *Ae. albopictus* control larvae (mean ± SD: *Cx. pipiens* = 1.64 ± 0.18 mm, *Ae. albopictus* = 1.36 ± 0.13 mm; *p*‐value = <.0001). Within *Cx. pipiens*, surviving control larvae were significantly larger from replicates held at 15°C, relative to those from 25°C (mean ± SD: 15°C = 1.66 ± 0.01 mm, 25°C = 1.60 ± 0.02 mm; *p*‐value = .0065). For *Ae. albopictus* surviving control larvae, there was no significant difference in length due to temperature (mean ± SD: 15°C = 1.35 ± 0.01 mm, 25°C = 1.36 ± 0.01 mm; *p*‐value = .4343).

**FIGURE 3:**
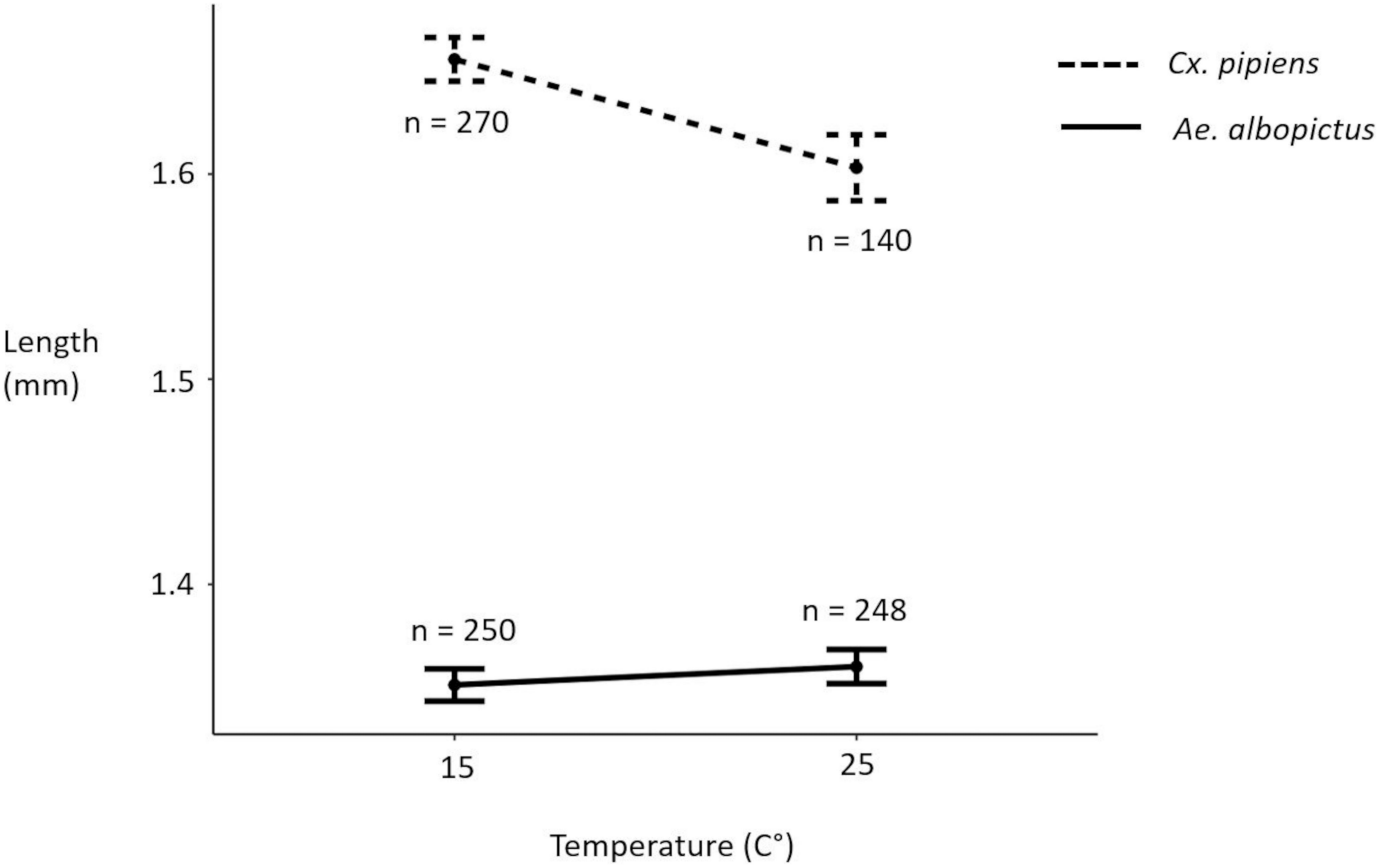
Interaction plot of relationship between length of surviving control larvae and temperature, modified by mosquito species (Error bars represent ± SE).

The larval length model with mosquito species as the only predictor had a similar AIC value (difference of <2) to the model that tested for interaction between mosquito species and temperature (Table S2). The results of the full model (Table 1) show that the interaction term was significant, indicating that the relationship between length of surviving control larvae and temperature was modified by mosquito species (Figure 3).

**TABLE 1:** Full linear model of length among surviving control larvae, allowing for interaction between temperature and mosquito species (*n* = 908).

Parameter	Estimate	Standard error	*p*‐value	Adjusted *R* ^2^
Intercept	1.338	0.028	<.0001	.458
Temperature (ref = 15°C)	0.001	0.001	.5158	
Mosquito species (*Ae. albopictus* = 0, *Cx. pipiens* = 1)	0.398	0.042	<.0001	
Temperature × Mosquito species	−0.006	0.002	.0035	

Further investigation of the larval lengths among only *Cx. pipiens* mosquitoes showed that the linear model with both egg raft and temperature as predictors had a similar AIC value (difference of <2) to the model that only included egg raft (Table S3). The results of the model that included temperature show that *Cx. pipiens* larval lengths significantly decrease with a 10°C increase in temperature, after controlling for natural variation in length due to egg raft effects (Table S4).

**FIGURE 4:**
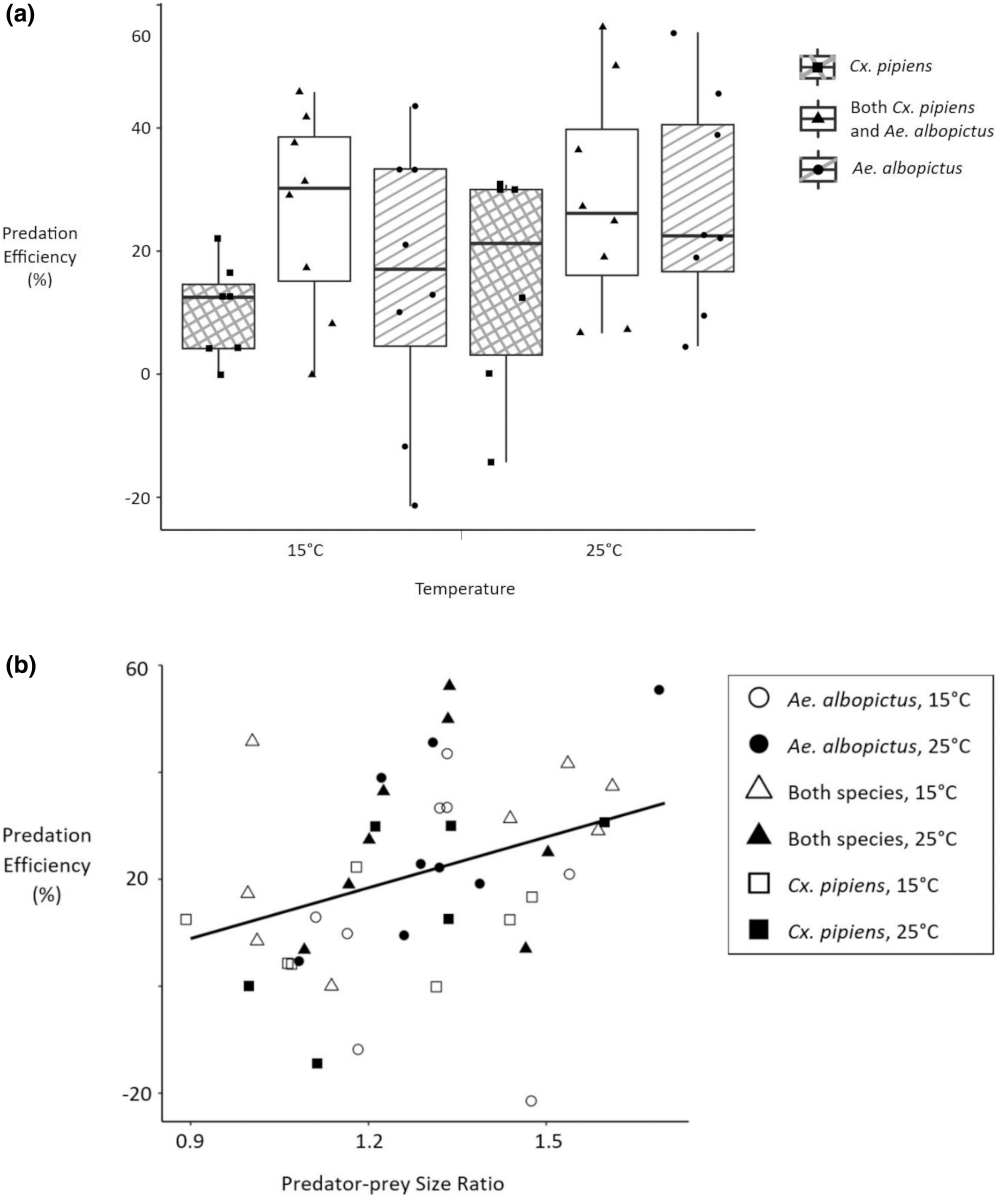
(a) Box plot of predation efficiencies by temperature and prey species composition (raw data points in the ‘jitter’ position). (b) Scatter plot of predation efficiency by predator–prey size ratio.

*Megacyclops viridis* lengths ranged from 1.5 to 2.5 mm (mean = 1.86, SD = 0.24). The predation efficiency model of best fit, based on AIC value, was the model that had predator–prey size ratio as the only predictor (Table S5). Adding temperature predictors to the model did not improve model fit (Table S5). Predator–prey size ratio was a significant predictor of predation efficiency (*p*‐value = .0276), and for each unit increase in size ratio, predation efficiency increased by approximately 32% (Table 2, Figure 4).

**TABLE 2:** Linear regression of predation efficiency by predator–prey size ratio (*n* = 45).

Parameter	Estimate	Standard error	*p*‐value	Adjusted *R* ^2^
Intercept	−19.56	17.92	.2810	.087
Predator–prey size ratio	31.64	13.88	.0276	

**FIGURE 5:**
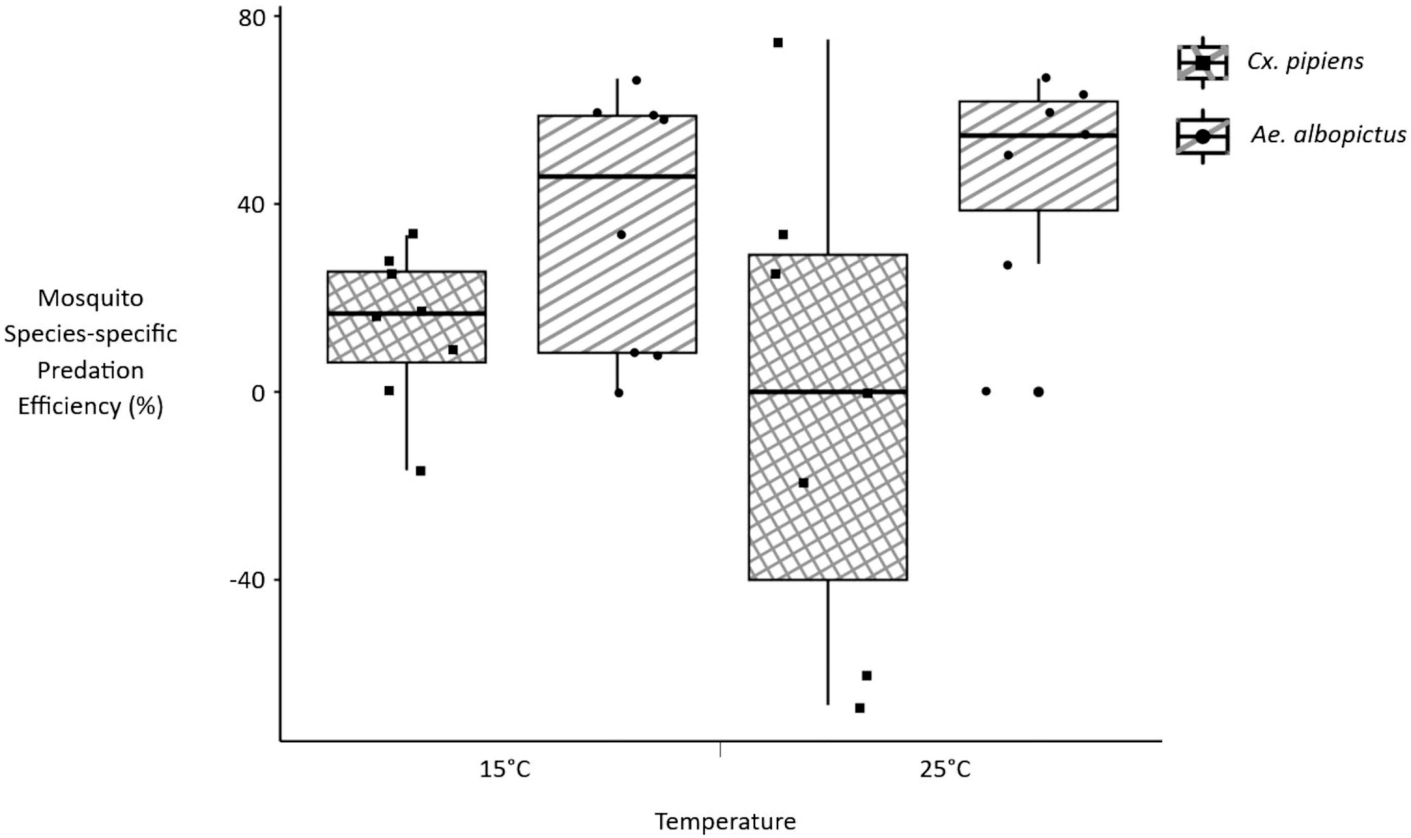
Box plot of mosquito species‐specific predation efficiencies in treatments that contained both *Culex pipiens* and *Aedes albopictus* larvae (raw data points in the ‘jitter’ position).

Among replicates that contained a single prey species (*n* = 29), there was not a significant difference in predation efficiency due to mosquito species (mean ± SD: *Cx. pipiens* = 12.4 ± 3.8%, *Ae. albopictus* = 21.2 ± 5.2%; *p*‐value = .1811). However, the difference in prey species‐specific predation efficiencies within replicates that contained both *Cx. pipiens* and *Ae. albopictus* (*n* = 15) was significantly different than zero (*p*‐value = .0107). On average, the *Ae. albopictus* predation efficiency was 34.5, 95% CI: (9.4, 59.7), percentage points higher than the *Cx. pipiens* predation efficiency in replicates that had both prey species (Figure 5).

## 4 DISCUSSION

I found that predator–prey size ratio is a significant predictor of predation efficiency for *M. viridis* cyclopoid copepods foraging on both invasive *Ae. albopictus* larvae and *Cx. pipiens* larvae native to Berkshire, UK (Table 2, Figure 4). Within treatments where both *Ae. albopictus* and *Cx. pipiens* larvae were offered to *M. viridis* together, the predation efficiency calculated for smaller *Ae. albopictus* prey was significantly higher than that which was calculated for larger *Cx. pipiens* prey (Figure 5). The large amount of variation in the predation efficiency values for *Cx. pipiens* prey at 25°C was mainly due to occasionally high background mortality among *Cx. pipiens* larvae at the higher temperature setting (Figure 5).

The survival of newly hatched *Cx. pipiens* larvae was significantly lower at 25°C than at 15°C. Similar negative impacts of maintenance at 25°C have previously been observed for wild‐caught UK mosquitoes of a different species, *Ae. detritus* (Lumley et al., 2018). Because the schedule of the experiment (Figure 1) was too short to allow for temperature to strongly affect development, the inverse relationship between temperature and size observed in *Cx. pipiens* larvae (Figure 3, Table S4) was most likely caused by higher mortality among larger *Cx. pipiens* larvae at 25°C. Based on these results, colonies of *Cx. pipiens* that are maintained at 25°C (Hernandez‐Triana et al., 2018; Manley et al., 2015) are likely to produce mosquitoes that are smaller than those found in natural UK populations. Lower colony maintenance temperatures, such as 22°C used for *Cx. pipiens* from Germany (Jourdan et al., 2016), or 23°C used for *Cx. pipiens* from the Netherlands (Manley et al., 2015), should also be used for *Cx. pipiens* from the United Kingdom. The low survival of *Cx. pipiens* larvae at 25°C also suggests that United Kingdom *Cx. pipiens* and *Ae. albopictus* might not share larval habitats as frequently as they have in regions at lower latitudes, such as Italy (Carrieri et al., 2003). Because *Cx. pipiens* larvae are known to inhabit both large permanent habitats (Amini et al., 2020; Lühken et al., 2015; Vinogradova, 2000), as well as container environments (Nikookar et al., 2017; Sulesco et al., 2015; Townroe & Callaghan, 2014; Verna, 2015), climate change may result in more frequent use of larger aquatic habitats, where water temperatures tend to be lower.

Predator–prey size ratio was a significant predictor of predation efficiency across all treatments (Table 2, Figure 4). Cyclopoid copepods *Macrocyclops distinctus*, *M. viridis*, and *Mesocyclops pehpeiensis* have all exhibited higher predatory ability against early instar *Ae. albopictus* larvae, relative to their predatory ability against larger late instar *Ae. albopictus* larvae (Dieng et al., 2002). Previous work has shown that patterns in prey size selection are based on maximising the energetic gain of the predator (Mittelbach, 1981). For copepods, the optimal predator‐to‐prey size ratio has been estimated at 18:1 (Hansen et al., 1994). Based on this size ratio, both *Cx. pipiens* and *Ae. albopictus* larvae are much larger than the optimal prey for *M. viridis*, and it is expected that *M. viridis* would prefer the smaller larvae. Within treatments that had both prey species, *Ae. albopictus* predation efficiency was about 34.5 percentage points higher than *Cx. pipiens* predation efficiency (Figure 5). While species‐specific larval behaviour may have also contributed to the higher vulnerability of *Ae. albopictus* (Kesavaraju et al., 2011), the results of my analyses suggest that its smaller size (Figure 3) played a significant role. In a similar experiment, where *Mesocyclops leuckarti* cyclopoid copepods were less effective against early instar *Ae. koreicus* prey than they were against early instar *Ae. albopictus* prey, the larger size of *Ae. koreicus* was provided as an explanation for the observed difference in predation efficiency (Baldacchino et al., 2017). It has previously been hypothesised that genus‐level morphological features of *Culex* larvae, such as long bristles, give copepod predators the ‘false impression’ that these larvae are too large (Marten et al., 1994). My results show that for copepod biocontrol agents that could be applied to larval habitats in South East England to help control *Ae. albopictus*, the larger size of *Cx. pipiens* will not be an illusion (Table 1, Figure 3).

Because *Aedes* mosquitoes are the costliest invasive taxon worldwide (Diagne et al., 2021), it is important to devise multi‐faceted ‘integrated vector control’ plans to keep mosquito populations as low as possible (Lacey & Orr, 1994). My findings show that the efficacy of *M. viridis* biocontrol agents against *Ae. albopictus* prey would not be hindered in the presence of native *Cx. pipiens* larvae. This information suggests that including scheduled applications of *M. viridis* copepods to artificial containers could be a beneficial component of vector control plans in South East England. Furthermore, it seems likely that the presence of *Cx. pipiens* in the same habitats where *Ae. albopictus* are likely to be found could aid control efforts by providing copepods with an additional food source in the absence of the invasive species (Murdoch & Bence, 1987).

## 5 LIMITATIONS

Some questions about levels of larval activity and interactions between *Ae. albopictus* and *Cx. pipiens* in the presence of a copepod predator remain unanswered because my experiments did not include close observation of the mosquito larvae during the 6 h period of predation. Among replicates that contained a single prey species, I did not observe a significant difference in predation efficiency between *Cx. pipiens* and *Ae. albopictus* prey, but when I compared prey species‐specific predation efficiencies from treatments where both mosquito prey species were present, I did observe a significant difference. These results are consistent with the findings of a previous study that examined copepod predation of *Cx. pipiens* and *Ae. aegypti* larvae. ‘There was no significant differences in mortality when *Ae. aegypti* and *Cx. pipiens* were tested separately, in contrast when both culicid species were simultaneously offered to copepod a selective consumption of *Ae. aegypti* larvae was registered’ (Micieli et al., 2002). While larval navigation behaviour varies across mosquito genera, most mosquito larvae exhibit some form of aggregation behaviour (Lutz et al., 2020). It is possible that when *Culex* and *Aedes* larvae are present in the same container, *Aedes* larvae are not able to aggregate as closely as they normally would, and this leaves the *Aedes* larvae more vulnerable to copepod predation. In future experiments, the incorporation of periodic photographs or video‐tracking software (Gonzalez et al., 2017) would be helpful for examining this hypothesis.

In addition, more observations of larvae during the 6 h period of predation would have allowed potential differences in antipredator behaviours between the two prey species to be documented. For example, *Cx. pipiens* responds to notonectid predator presence by reducing its movement, and the antipredator response of *Cx. pipiens* is stronger than that of *Ae. aegypti* (Sih, 1986). Reduced movement by *Cx. pipiens* larvae would likely result in lower copepod predation efficiency within this prey group.

Finally, these short‐term laboratory experiments may not be realistic enough to fully support field applications of *M. viridis* copepods to artificial container environments in South East England. Support for using cyclopoid copepods as biocontrol agents in the United Kingdom could be strengthened by a long‐term field study based in southern France, where *Ae. albopictus* populations have already established.

## AUTHOR CONTRIBUTION

**Marie C. Russell:** Conceptualization (lead); data curation (lead); formal analysis (lead); funding acquisition (lead); investigation (lead); methodology (lead); project administration (lead); resources (lead); software (lead); supervision (lead); validation (lead); visualization (lead); writing – original draft (lead); writing – review and editing (lead).

## CONFLICT OF INTEREST STATEMENT

The author has declared no competing interest.

## Supporting information


Table S1–S5.

## Data Availability

Study data are accessible from the Dryad Digital Repository (https://doi.org/10.5061/dryad.vt4b8gtwv).
