# Supplementary material for: A difference in larval mosquito size allows a biocontrol agent to target the invasive species
Source: Ecol Evol. 2023 Jul 10;13(7):e10294. doi: 10.1002/ece3.10294 (PMC10333674; doi:10.1002/ece3.10294)
Supplement: Supplementary file 1 — Table S1–S5. [file ECE3-13-e10294-s001.docx]

**Appendix**

Table S1. GenBank BLAST Percent Identity of Sequences to Accession FN395181.1 “*Culex pipiens pipiens* mitochondrial COI gene for cytochrome oxidase subunit I, isolate Iksha3” (length = 1542 bp)

| **Egg Raft ID#** | **Primer Name** | **Length (bp)** | **% Query Cover** | **% Identity** | **E value** | **Bit-score** |
| --- | --- | --- | --- | --- | --- | --- |
| 1 | C1-J-2183 | 849 | 96 | 100.00 | 0.0 | 1507 |
| 1 | TL2-N-3014 | 850 | 97 | 99.76 | 0.0 | 1517 |
| 4 | C1-J-2183 | 846 | 96 | 99.88 | 0.0 | 1500 |
| 4 | TL2-N-3014 | 848 | 98 | 99.28 | 0.0 | 1511 |
| 8 | C1-J-2183 | 845 | 96 | 100.00 | 0.0 | 1507 |
| 8 | TL2-N-3014 | 847 | 97 | 99.88 | 0.0 | 1511 |
| 10 | C1-J-2183 | 849 | 95 | 100.00 | 0.0 | 1504 |
| 10 | TL2-N-3014 | 838 | 98 | 99.64 | 0.0 | 1511 |
| 11 | C1-J-2183 | 854 | 95 | 99.88 | 0.0 | 1507 |
| 11 | TL2-N-3014 | 838 | 97 | 100.00 | 0.0 | 1506 |
| 16 | C1-J-2183 | 842 | 96 | 100.00 | 0.0 | 1504 |
| 16 | TL2-N-3014 | 841 | 98 | 99.64 | 0.0 | 1511 |
| 18 | C1-J-2183 | 839 | 96 | 100.00 | 0.0 | 1493 |
| 18 | TL2-N-3014 | 841 | 98 | 99.64 | 0.0 | 1511 |
| 19 | C1-J-2183 | 842 | 96 | 99.88 | 0.0 | 1498 |
| 19 | TL2-N-3014 | 842 | 97 | 99.88 | 0.0 | 1506 |

Table S2. Length of surviving control larvae model selection, ranked by AIC.

| **Parameters** | **Degrees of Freedom** | **Adjusted R^2^** | **AIC** |
| --- | --- | --- | --- |
| Mosquito species | 906 | 0.453 | -813.16 |
| Mosquito species, Temperature (ref = 15°C), Mosquito species x Temperature | 904 | 0.458 | -812.45 |
| Mosquito species, Temperature | 905 | 0.454 | -810.89 |

Table S3. Length of surviving control *Cx. pipiens* larvae model selection, ranked by AIC.

| **Parameters** | **Degrees of Freedom** | **Adjusted R^2^** | **AIC** |
| --- | --- | --- | --- |
| Egg raft, Temperature (ref = 15°C) | 401 | 0.699 | -655.65 |
| Egg raft | 402 | 0.687 | -655.03 |
| Egg raft, Temperature, Egg raft x Temperature | 394 | 0.729 | -531.04 |
| Temperature | 408 | 0.017 | -239.78 |

Table S4. Linear regression of larval length among surviving control *Cx. pipiens* by egg raft and temperature (n = 410).

| **Parameter** | **Estimate** | **Standard Error** | **p-value** | **Adjusted R^2^** |
| --- | --- | --- | --- | --- |
| Intercept | 1.427 | 0.026 | <0.0001 | 0.699 |
| Temperature (ref = 15°C) | -0.005 | 0.001 | <0.0001 |  |
| Egg raft 2 (ref = Egg raft 1) | 0.139 | 0.021 | <0.0001 |  |
| Egg raft 3 (ref = Egg raft 1) | 0.235 | 0.023 | <0.0001 |  |
| Egg raft 4 (ref = Egg raft 1) | 0.262 | 0.020 | <0.0001 |  |
| Egg raft 5 (ref = Egg raft 1) | 0.457 | 0.022 | <0.0001 |  |
| Egg raft 6 (ref = Egg raft 1) | 0.522 | 0.022 | <0.0001 |  |
| Egg raft 7 (ref = Egg raft 1) | 0.410 | 0.019 | <0.0001 |  |
| Egg raft 8 (ref = Egg raft 1) | 0.288 | 0.020 | <0.0001 |  |

Table S5. Predation efficiency model selection, ranked by AIC.

| **Parameters** | **Degrees of Freedom** | **Adjusted R^2^** | **AIC** |
| --- | --- | --- | --- |
| Predator-prey size ratio | 43 | 0.087 | 385.32 |
| Copepod length | 43 | 0.022 | 388.40 |
| Copepod body mass | 43 | 0.008 | 389.06 |
| Predator-prey size ratio, Temperature | 42 | 0.094 | 388.92 |
| Predator-prey size ratio, Temperature, Predator-prey size ratio x Temperature | 41 | 0.100 | 394.53 |
